# The Deep Learning-Crop Platform (DL-CRoP): For Species-Level Identification and Nutrient Status of Agricultural Crops

**DOI:** 10.34133/research.0491

**Published:** 2024-10-04

**Authors:** Mohammad Urfan, Prakriti Rajput, Palak Mahajan, Shubham Sharma, Haroon Rashid Hakla, Verasis Kour, Bhubneshwari Khajuria, Rehana Chowdhary, Parveen Kumar Lehana, Namrata Karlupia, Pawanesh Abrol, Lam Son Phan Tran, Sikander PAL Choudhary

**Affiliations:** ^1^ Crop Physiology Laboratory, Department of Botany, University of Jammu, Jammu 180006, India.; ^2^ Department of Computer Science & Engineering, Central University of Jammu, Jammu 181143, India.; ^3^ Department of Electronics, University of Jammu, Jammu 180006, India.; ^4^ Department of Computer Science & IT, University of Jammu, Jammu 180006, India.; ^5^ Institute of Genomics for Crop Abiotic Stress Tolerance, Department of Plant and Soil Science, Texas Tech University, Lubbock, TX 79409, USA.

## Abstract

Precise and timely detection of a crop’s nutrient requirement will play a crucial role in assuring optimum plant growth and crop yield. The present study introduces a reliable deep learning platform called “Deep Learning-Crop Platform” (DL-CRoP) for the identification of some commercially grown plants and their nutrient requirements using leaf, stem, and root images using a convolutional neural network (CNN). It extracts intrinsic feature patterns through hierarchical mapping and provides remarkable outcomes in identification tasks. The DL-CRoP platform is trained on the plant image dataset, namely, Jammu University-Botany Image Database (JU-BID), available at https://github.com/urfanbutt. The findings demonstrate implementation of DL-CRoP—cases A (uses shoot images) and B (uses leaf images) for species identification for *Solanum lycopersicum* (tomato), *Vigna radiata* (Vigna), and *Zea mays* (maize), and cases C (uses leaf images) and D (uses root images) for diagnosis of nitrogen deficiency in maize. The platform achieved a higher rate of accuracy at 80–20, 70–30, and 60–40 splits for all the case studies, compared with established algorithms such as random forest, K-nearest neighbor, support vector machine, AdaBoost, and naïve Bayes. It provides a higher accuracy rate in classification parameters like recall, precision, and F1 score for cases A (90.45%), B (100%), and C (93.21), while a medium-level accuracy of 68.54% for case D. To further improve the accuracy of the platform in case study C, the CNN was modified including a multi-head attention (MHA) block. It resulted in the enhancement of the accuracy of classifying the nitrogen deficiency above 95%. The platform could play an important role in evaluating the health status of crop plants along with a role in precise identification of species. It may be used as a better module for precision crop cultivation under limited nutrient conditions.

## Introduction

Recent years have seen unprecedented applications of machine learning (ML) to several problems in computer vision like semantic segmentation [1], facial recognition [2], and image annotation [3]. These advancements have provided a platform for large labeled datasets, which have provided computing technologies with high-performance and robust deep-learning (DL) algorithms [4,5]. From past experiences, conventional ML techniques have struggled to process the data in their natural raw form. To overcome this issue, generic nonlinear methods of feature extractions like multi-stage hand-tuned pipelines and kernel methods have been developed. These techniques of extraction and discriminative classifiers are now widely employed. Although these classifiers could improve ML precision, the aforementioned generic features often limit the learner’s ability to draw concrete conclusions from the training examples [4].

In such a scenario, DL has emerged as a robust and reliable ML technique. It works on the concept of neural networking in modeling thereby solving complex problems in areas of animal and plant sciences [6–8]. The DL techniques have revolutionized computer vision tasks through automated prediction analysis. It is a successful tool in the image-based identification of large data of plants [9]. Further new approaches have been developed in DL systems to extract distinct leaf image features along with the application of a classifier for species identification in animals and plants. Besides the DL application in agriculture sciences, DL is widely used for multiple areas serving mankind such as visual object recognition and detection, speech recognition, genomics, and drug discovery [10,11]. More recently, DeepSecE, a type of DL, has been successfully used in gram-negative bacteria for the multiclass prediction of secreted proteins [12]. DeepSecE was able to achieve a 0.883 macro-average accuracy of 5-fold cross-validation in the prediction of proteins secreted using a pretrained protein language model and transformer [12]. A soft crawling robot with multimodal locomotion has been developed, inspired by the crawling *Drosophila larvae* [13]. These soft crawling robots could represent a true fusion of biodesign and application of DL in performing multimodal inspection of areas with these robots [13]. Another diverse application includes a combination of DL and soundscapes for tracking the recovery of biodiversity in tropical forests [14]. The acoustic index model and a bird community composition achieved with a convolutional neural network (CNN) showed strong correlations with the restoration parameters by adjusting *R*^2^ to 0.62 and 0.69, respectively [14]. With the ever-increasing human population, early and precise detection of crop systems prone to or suffering from pest diseases and nutrient deficiencies is of utmost importance [11]. In general, the DL is based on several algorithms like CNNs, generative adversarial networks (GANs), recurrent neural networks (RNNs), long short-term memory networks (LSTMs), self-organizing maps (SOMs), radial basis function networks (RBFNs), multilayer perceptrons (MLPs), restricted Boltzmann machines (RBMs), deep belief networks (DBNs), and autoencoders. Among these algorithms, the CNN-based DL is gaining scientific attention for its problem-solving and highest computational power [10–14]. The CNN-based DL has nested in depth into the empowerment of the agricultural domain with effective decision-making in crop management, species identification, health identification, etc. [10–14]. Addressing nutrient deficiencies is critical for global food security, as these deficiencies can reduce crop yields by up to 30% to 35% [15]. Nitrogen, a vital nutrient, is crucial for amino acids, proteins, nucleic acids, and chlorophyll synthesis in plants. Its deficiency alone can result in stunted growth, smaller leaf size, and potentially plant death, which in turn can lead to substantial yield losses and increased vulnerability to diseases [16]. Various visually observable plant traits like leaf color, number of leaves, leaf area, root–shoot ratio, lateral root growth, and plant height are affected by different levels of nitrogen. These traits can be used for the early detection and management of nitrogen deficiency, which is crucial for maintaining crop yields and securing global food supplies [17]. Besides the conventional CNN modules, the latest computer vision systems have been reported using multi-head attention (MHA) mechanisms [18,19]. Although MHA has been successfully used in classifying human facial images, its applications in the diagnosis of nutrient deficiency in crops remain unexplored.

In this research, a novel CNN-based DL framework has been developed. It has been successful for plant classification and nutrient deficiency detection in crop plants. The DL experiments on *Zea mays* L. (maize), *Vigna radiata* ( Vigna), and *Solanum lycopersicum* L. (tomato) could automatically identify plants based on image datasets. The working efficiency of the present CNN framework called Deep Learning-Crop Platform (DL-CRoP) was compared with other models of DL as well.

## Results

The experimental work for the proposed framework has been performed on an Intel i7 8750H CPU having NVIDIA, GPU, and the GeForce GTX model 1050Ti. The CNNs have been implemented using MATLAB R2019a on Windows 11 with vision architectures. The performance of InceptionResNetV2 as a backbone network for transfer learning the crop species and plant health identification was analyzed with 4 case studies (A to D) with 3 and 2 class-based classification scenarios, respectively.

### Case A: Species identification—stem image database

**Fig. 1.**
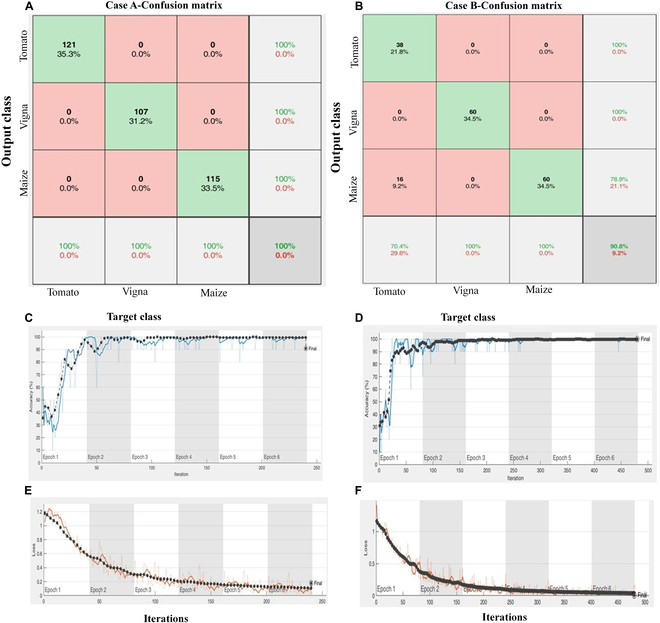
Prediction of species-level identification with DL-CRoP. (A) Case A represents the DL-CRoP trained stem image dataset in matrix mode with 100% accuracy achieved in classifying the stems of tomato, Vigna, and maize at the species level. (B) Case B represents DL-CRoP trained leaf image dataset in matrix mode 90.8% accuracy achieved in classifying the leaf images of tomato, Vigna, and maize at the species level. (C) increase in accuracy (%) for the correct detection of stem images of tomato, Vigna, and maize. (D) increase in accuracy (%) for the correct detection of leaf images of tomato, Vigna, and maize. (E) Reduction in error loss with increase in the number of stem input image number in the detection of stems of tomato, Vigna, and maize at the species level. (F) Reduction in error loss with increase in the number of leaf input images in the detection of the leaf of tomato, Vigna, and maize.

Initially, the proposed DL-CRoP architecture and training methodology was thoroughly applied to case A where the classification problem was a 3-class-based identification of crop plant shoot images. The confusion matrix generated for the current study showed zero misclassified class instances that occurred in the stem identification of tomato, Vigna, and maize (Fig. 1A). Hence, the model outperformed in case study A by justifying 100% model accuracy. The error in stem image-based identification of different plant species, namely, tomato, Vigna, and maize, was reduced with a consistent increase in the number of input images as shown in error loss and accuracy (Fig. 1C and E). DL-CRoP for case study A yielded a precision (0.901), recall (0.929), and F1 score (0.915) (Table 1). The model achieved an early stopping at the 6th epoch with an accuracy level of 100%. The graphical accuracies and loss profiles for 3 classes of classification based on shoot images—tomato, Vigna, and maize for case study A—are shown in Fig. 1C and E. Evident from the curves, the proposed framework perfectly fitted well to identify shoot images of different species. It is pertinent to mention that the quality of the images captured with a size greater than 3 MB could enhance the identification capability of the model to the maximum, thereby achieving 100% accuracy.

**Table 1.** Classification parameters: Precision, recall, and F1 score for case study A (stem image database) of tomato, Vigna, and maize used for stem-based species-level identification, case study B (leaf image database) of tomato, Vigna, and maize used for leaf image level species identification, case study C (leaf image database) of maize plants subjected to normal and low nitrogen condition for the detection of nutrient (nitrogen) deficiency in maize leaf, and case study D (root image database) of maize plants subjected to high and low nitrogen condition for the detection of nitrogen deficiency in maize roots.

Case study	Classification parameters		
	Precision	Recall	F1 score
Case A: Leaf species	0.9012	0.9298	0.9153
Case B: Stem species	1	1	1
Case C: Leaf deficiency	0.9259	0.9201	0.9230
Case D: Root deficiency	0.676	0.685	0.681

### Case B: Species identification—leaf image database

For the same configuration details, the proposed model was further tested on case B with leaf images. The proposed DL-CRoP for leaf image identification achieved an accuracy of 90.8% while training the network for 50 epochs for 3-class scenarios. DL-CRoP could identify tomato, Vigna, and maize using the leaf images. The confusion matrix showed that 9.2% of misclassified class instances occurred in the leaf image identification of tomato, Vigna, and maize (Fig. 1B). Hence, the model in case study B provided 90.8% accuracy. The error in leaf image-based identification of tomato, Vigna and maize was reduced with a consistent increase in the number of input images as shown in error loss and accuracy (Fig. 1D and F). A leaf depicts prominent features in crop plants, and the performance of the proposed DL-CRoP framework demonstrated by the confusion matrix showed potential utility in various domains of agriculture, for instance, identifying species based on leaf patterns. DL-CRoP for case study B had better precision (1), recall (1), and F1 score (1) compared to that for case A (Table 1).

### Case C: Leaf nutrient (nitrogen) deficiency

**Fig. 2.**
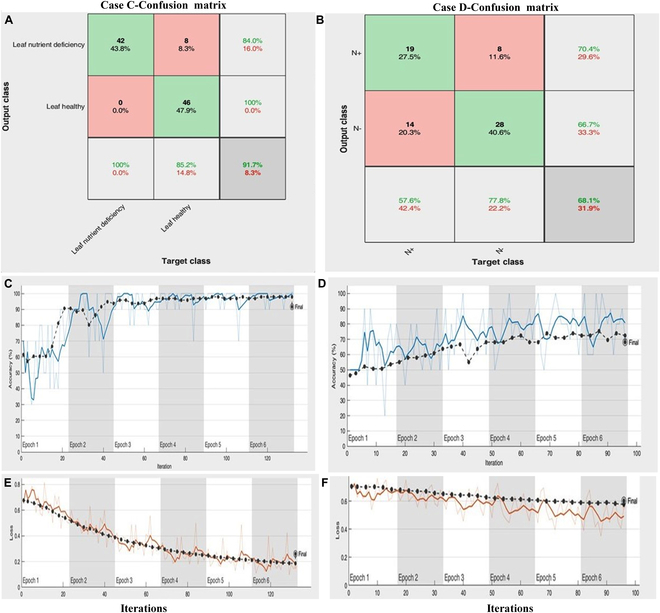
Prediction of nutrient deficiency in leaf and root of maize. (A) Case C represents DL-CRoP trained on the leaf image dataset in matrix mode with 91.7% accuracy achieved in classifying the leaf of maize plants deficient in nitrogen. (B) Case D represents the root image dataset in matrix mode with a DL-CRoP 68.1% accuracy achieved in classifying the root images of maize plants deficient in nitrogen. (C) Increase in accuracy (%) for the correct detection of leaf images of maize plants deficient in nitrogen. (D) Increase in accuracy (%) for the correct detection of root images of maize plants deficient in nitrogen. (E) Reduction in error loss with increase in the number of input leaf image number in the detection of maize leaf deficient in nitrogen. (F) Reduction in error loss with increase in the input root image number in the detection of maize roots deficient in nitrogen.

The proposed framework of DL-CRoP was further extended for leaf health identification by estimating the deficiency of nutrients (nitrogen) in maize leaves. The model proposed achieved an accuracy of 91.7% for 2-class scenarios: high nitrogen (healthy leaf) and low nitrogen (nutrient-deficient leaf), respectively, validated through the graph convergence during the training of the model. The confusion matrix generated for the case study is represented (Fig. 2A). The error in leaf image-based identification of maize leaf deficient in nitrogen was reduced to 8.3% with a consistent increase in the number of input images as shown in error loss and accuracy (Fig. 2C and E). For case study C, DL-CRoP showed a precision (0.925), recall (0.920), and F1 score (0.923), which were lower than for case study B but higher than for case study A except for recall (Table 1).

### Case D: Root nutrient (nitrogen) deficiency

The DL algorithms for the identification of root-based nutrient status in crop plants are the least explored. The present case study D offers an out-of-the-box solution for identifying the nutrient status of a crop root using the DL-CRoP platform. For case study D, healthy and nitrogen-deficient maize root identification, DL-CRoP achieved an accuracy of 68.1% for 2-class scenarios: high nitrogen (N+) and low nitrogen (N−) (Fig. 2B). The root image structure was very similar for both classes, with the distinguishing feature being the length of the root. Many images were mislabeled with low nitrogen (N−), and the model was not able to generalize the root health identification to the highest level of accuracy (i.e., 100%). Extracting distinguishing features was a bit challenging task. The confusion matrix generated had many misclassified values along with a true positive (TP) and a true negative (TN) (Fig. 2B). The error in root image-based identification of maize root deficient in nitrogen was reduced with a consistent increase in the number of input images as shown in error loss and accuracy (Fig. 2D and F). For case study D, DL-CRoP was much lower in terms of precision (0.676), recall (0.685), and F1 score (0.681) when compared to other case studies (Table 1). The lowest accuracy level of 68.1% achieved in DL-CRoP may be attributed to similar root characteristics of maize roots under normal and nitrogen-deficient conditions. DL-CRoP used the root length and branching pattern as distinguishing features of maize roots under normal and nitrogen-deficient conditions. The potential reasons for DL-CRoP’s lesser accuracy may be attributed to the lesser striking difference in the root growth attributes between 2 classes, i.e., normal and nitrogen deficient; DL-CRoP achieved a lesser accuracy compared to case C. To reduce the discrepancy of DL-CRoP for case study D, root characteristics other than root length such as root branching pattern and root hair distribution may also be considered. However, taking higher-resolution root image data will be a challenging task to minimize this discrepancy. Furthermore, increasing the number of root image datasets can also improve the accuracy of DL-CRoP in terms of root nutrient diagnosis.

### DL-CRoP at a glance

Observation revealed that the N deficiency features were best extracted from maize leaf images compared to root images. The superior performance of the Inception net model could be attributed to its ability to perform convolutions of millions of images at multiple stages. It filters sizes while pooling within a single layer. This model’s increased depth compared to others helps reduce internal covariate shifts, resulting in more stable and accurate outcomes. Training the model took longer in case study B than in case studies A, C, and D. To ensure a meaningful representation of model performance and analysis across all case studies, 5 different batch sizes and 6 epochs were selected for training. The loss function graphs for case studies A to D demonstrated a decrease in the loss factor of the training process as the number of epochs increased. Further, it was noted that at 50 epochs, less fluctuation occurred in the loss function and was more stable and converged. The variation in percentage accuracy could be due to the different case studies in the amount of image similarity and plant qualitative and quantitative traits in different species or the same species under different conditions. The justification of performance accuracy of case studies A to D was confirmed using confusion matrices (Figs. 1 and 2). The matrices of various case studies provide quantitative and predictive results for image identification. In the confusion matrix, the diagonals indicate the number of correctly predicted observations, meaning that the predicted class matched the actual class, while the rest of the observations represent misclassifications. The rows of the matrix represented instances of the predicted class, and the columns correspond to the instances of the actual class. The matrix includes an additional row and column to represent accuracy and error rates. Specifically, the first row contains values for TP, false positives (FP)/type I error, and accuracy, while the second row includes values for false negatives (FN)/type II error, TN, and accuracy.

### DL-CRoP vis-à-vis other algorithms

**Fig. 3.**
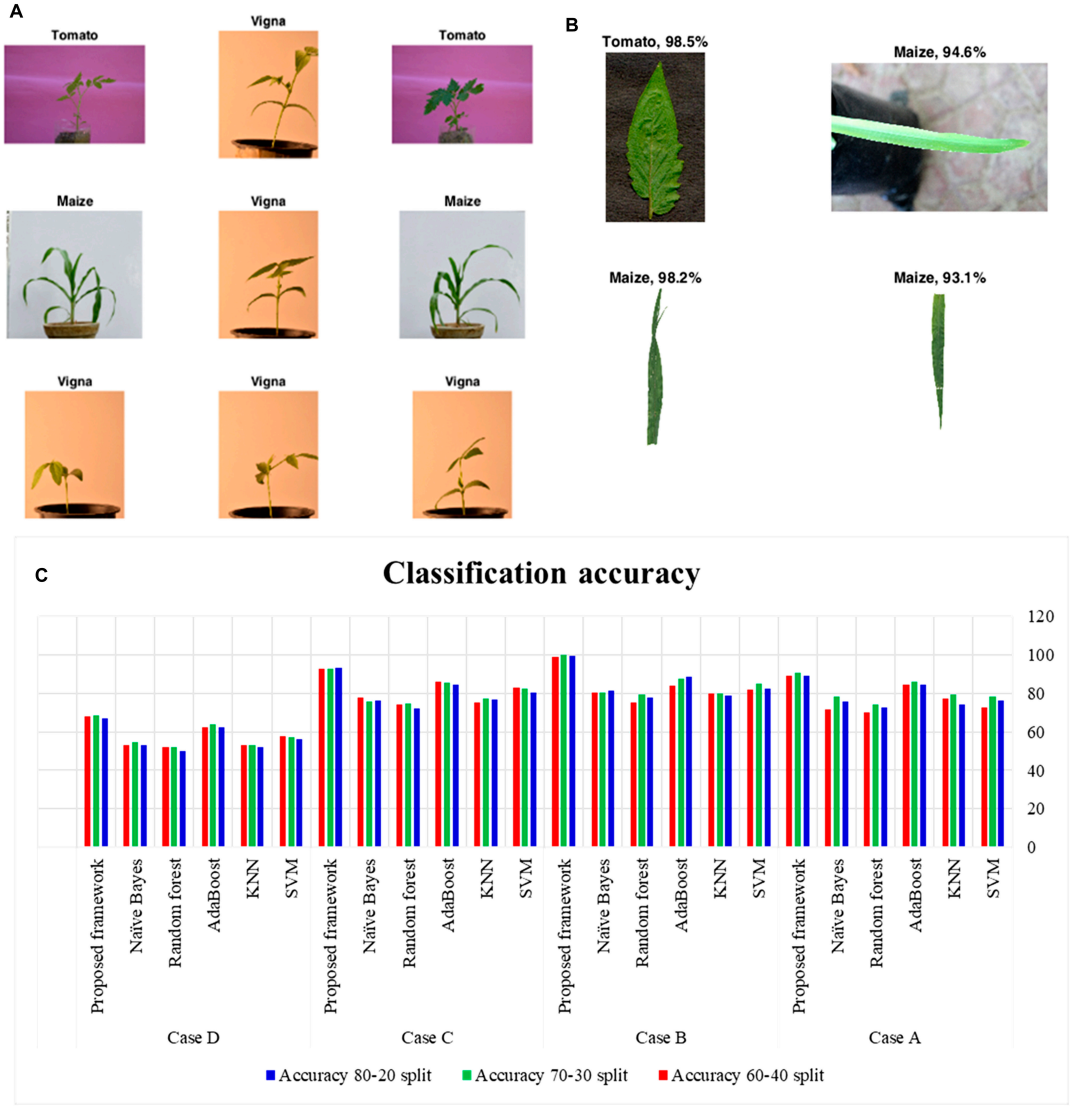
Classification accuracy of proposed InceptionResNetV2-based DL-CRoP. (A) Representative stem images of tomato, Vigna, and maize, used for CNN, (B) showing accuracy (%) in the correct detection of leaf images of tomato (98.5%) and maize (94.6%) for leaf image-based detection of tomato and maize, and the leaf of maize in normal (98.2%) and nitrogen-deficient condition (93.1%). (C) Classification accuracy, case study A (stem image database) of tomato, Vigna, and maize used for stem-based species-level identification, case study B (leaf image database) of tomato, Vigna, and maize used for leaf image level species identification, case study C (leaf image database) of maize plants subjected to normal and low nitrogen condition for the detection of nutrient (nitrogen) deficiency in maize, and case study D (root image database) of maize plants subjected to high and low nitrogen condition for the detection of nitrogen deficiency in maize roots. All these case studies were analyzed using naïve Bayes, random forest, AdaBoost, KNN, and SVM along with DL-CRoP (proposed framework). All these algorithms were compared with DL-CRoP of the current study and were tested for accuracy at 80–20, 70–30, and 60–40 splits. The DL-CRoP showed better accuracy in all the case studies compared to the other algorithms employed.

**Fig. 4.**
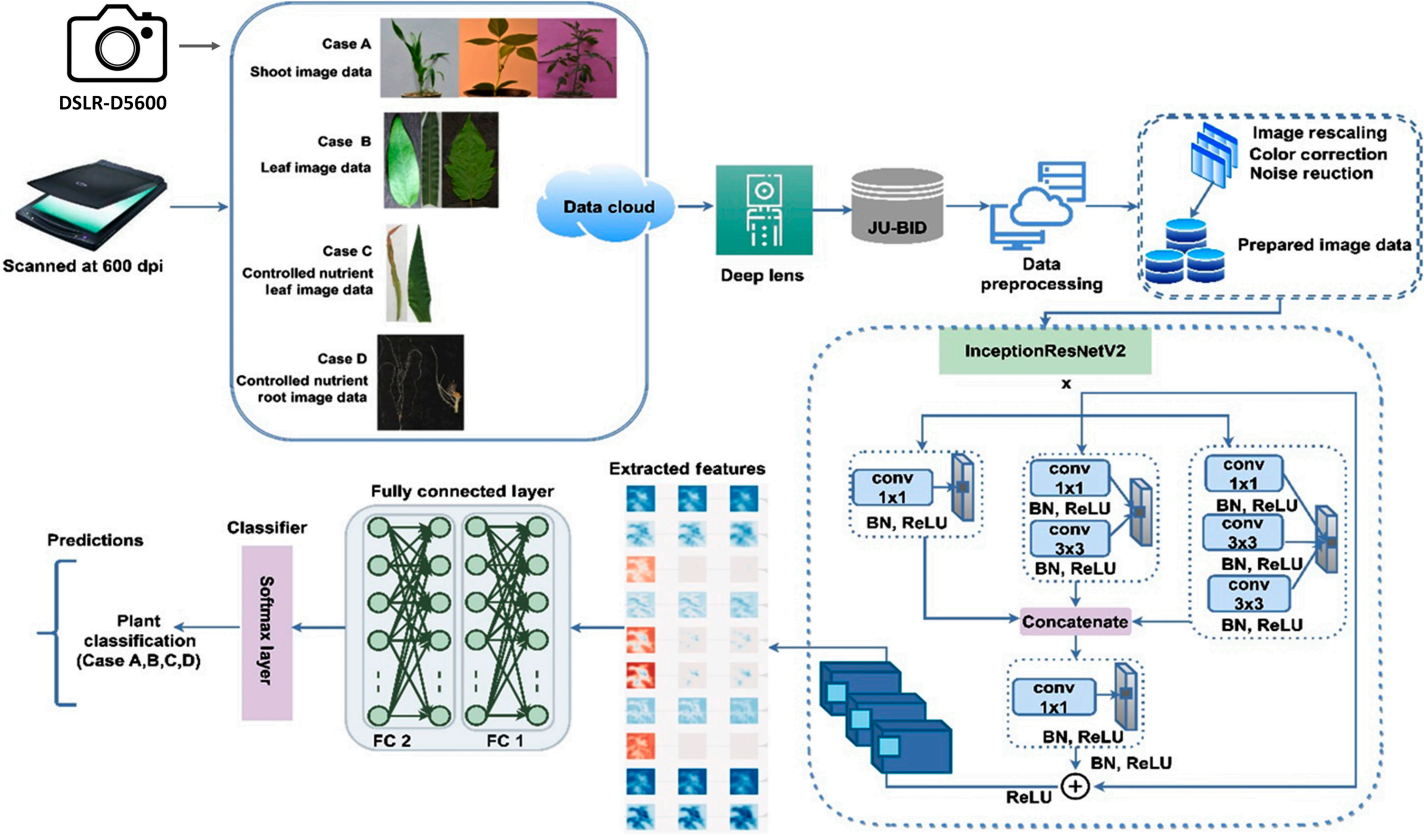
DL-CRoP architecture and training model. The architecture and training model of DL-CRoP included steps of data collection using a DSLR camera (Nikon, D5600) and a scanner (Epson Perfection V850 Pro Scanner), data processing, and model training followed by feature extraction—the data processing comprised image rescaling, color correction, and noise reduction. The processed data were applied for model training. The selected model during the present study was the InceptionResnetV2, composed of different convolution layers. The feature extraction was done through connected different convolution layers. The prediction and results of each case study, namely, case A (stem images of tomato, maize, and Vigna), case B (leaf images of tomato, maize, and Vigna), case C (maize leaf images of plants subjected to high and low nitrogen conditions), and case D (maize root images of plants subjected to high and low nitrogen condition) of trained data were made using Softmax layer and led to the prediction of different case studies. BN, batch normalization; FC, fully connected layer; conv, convolutional layer.

**Fig. 5.**
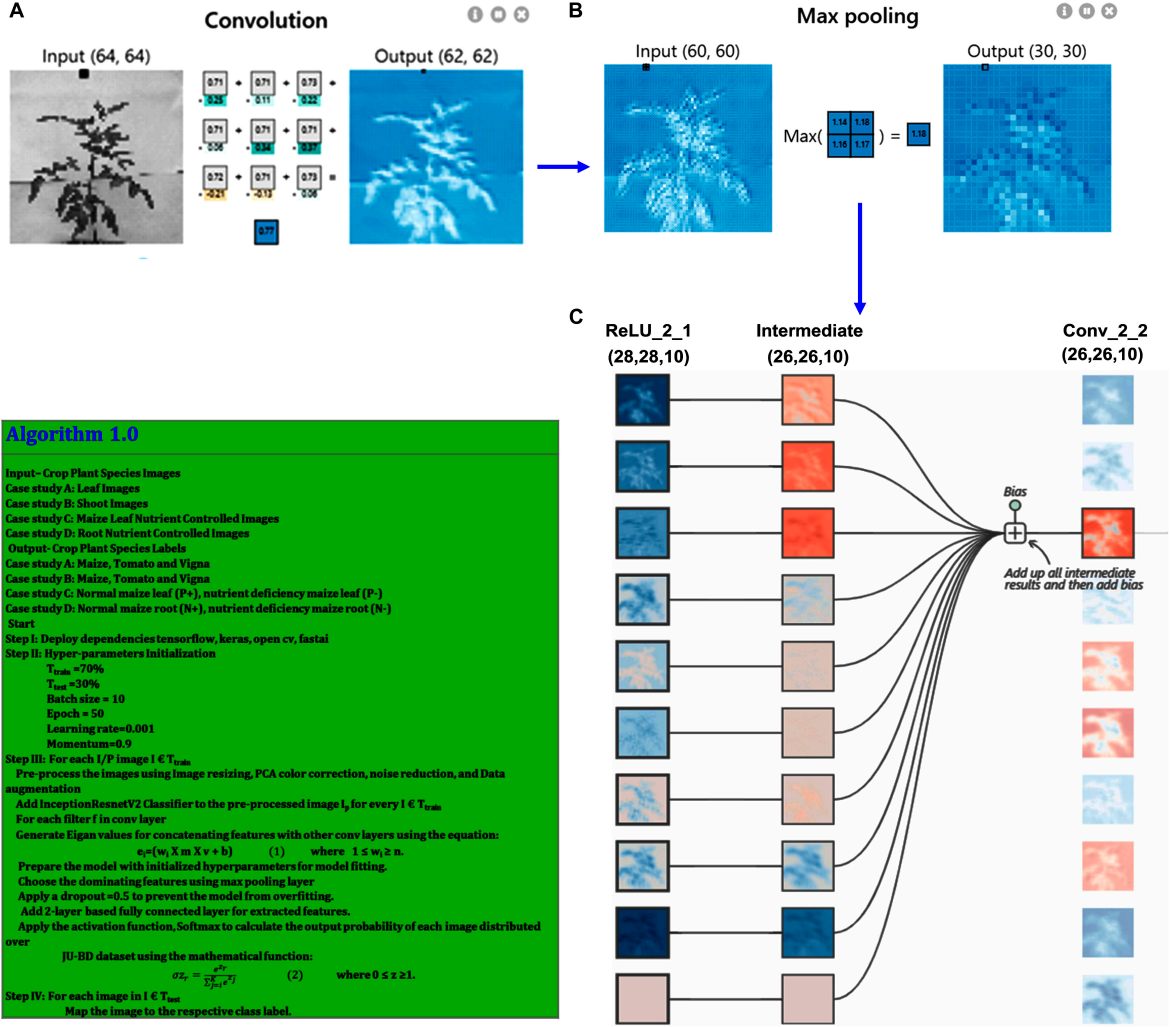
Flow diagram of DL-CRoP. (A) Image acquisition of a representative tomato plant 60 d old grown in a pot showing input and output of convolution step. (B) Max pooling with input and output data. (C) Data processing using convolutional layer, intermediate, and ReLU. Algorithm 1.0 used for image processing is shown in the green colored box.

**Fig. 6.**
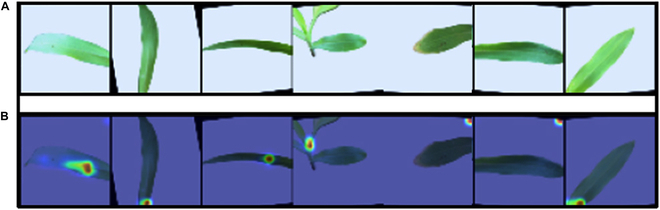
Attention map for leaf nitrogen diagnosis. The attention map shows how much attention different areas of a leaf image received during analysis. It indicates the important regions of maize leaf showing typical symptoms of nitrogen deficiency. (A) Input images. (B) Region marked by MHA.

A comparison of DL-CRoP with other algorithms in terms of classification accuracy was achieved for case studies A to D. The same JU-BID dataset at 80–20, 70–30, and 60–40 training–testing split was used for support vector machine (SVM), K-nearest neighbor (KNN), AdaBoost, naïve Bayes, and random forest. DL-CRoP on case A proved better than other algorithms and showed a higher classification accuracy of 90.45% compared to 78.27%, 79.44%, 86.25%, 74.11%, and 78.27% of SVM, KNN, AdaBoost, random forest, and naïve Bayes, respectively, at 70:30 training split ratios (Fig. 3 and Table 2). The classification accuracy of DL-CRoP for case study B was 100%, and none of the other algorithms could achieve this level of accuracy at 80:20, 70:30, and 60:40 training split ratios (Fig. 3 and Table 2). For case study C, the classification accuracy of DL-CRoP was 93.21%, 92.54%, and 92.69% at 80:20, 70:30, and 60:40 split ratios, respectively. This level of classification accuracy achieved by DL-CRoP for case study C was higher compared to all other algorithms at all the training split ratios (Fig. 3 and Table 2). Case study D offered a challenging task as root image-based identification of plant nutrient status is least explored with a DL algorithm. In this case, the classification accuracy of DL-CRoP was 66.91%, 68.54%, and 68.04% at 80:20, 70:30, and 60:40 training split ratios, respectively. Although the classification accuracy achieved by DL-CRoP for case study D was lowest compared to case studies A, B, and C, this classification accuracy was higher compared to other algorithms tested for case study D (Fig. 3 and Table 2). From this, it was evident that DL-CRoP (InceptionResNetV2-based CNN model) could outperform existing SVM, KNN, AdaBoost, random forest, and naïve Bayes algorithms. A simpler outlay of DL-CRoP makes it an exciting platform to predict the species-level identification and nutrient status of a crop plant in a highly precise and accurate manner (Figs. 4 and 5).To further evaluate the case study C accuracy, besides InceptionResNetV2, the MHA-based VGG-16 architecture was also investigated. The results obtained are presented in Fig. 6. The results demonstrate that MHA can precisely mark the leaf part, typically showing nitrogen deficiency in the attention map obtained after pooling layer 4. The MHA-based architecture was able to enhance the accuracy of the prediction above 95%, as compared to the accuracy of the InceptionResnet-v2, which was 91.7%.

**Table 2.** Comprehensive performance analysis of classification accuracy using machine learning models of SVM, KNN, AdaBoost, random forest, and naïve Bayes, compared with DL-CRoP (proposed module). The case studies used for the current study included case study A (stem image database) of tomato, maize, and Vigna used for stem-based species-level identification, case study B (leaf image database) of tomato, maize, and Vigna used for leaf image level species identification, case study C (leaf image database) of maize plants subjected to normal and low nitrogen condition for the detection of nutrient (nitrogen) deficiency in maize leaf, and case study D (root image database) of maize plants subjected to high and low nitrogen condition for the detection of nitrogen deficiency in maize roots with different split ratios (80–20, 70–30, and 60–40).

Case study	Algorithm	Accuracy		
		80–20 split	70–30 split	60–40 split
Case A	SVM	76.34	78.27	72.50
	KNN	74.19	79.44	77.27
	AdaBoost	84.44	86.25	84.51
	Random forest	72.84	74.11	70.23
	Naïve Bayes	75.82	78.27	71.50
	DL-CRoP	88.87	90.45	89.23
Case B	SVM	82.44	85.24	81.80
	KNN	79.01	80.01	79.77
	AdaBoost	88.70	87.77	84.14
	Random forest	77.89	79.16	75.40
	Naïve Bayes	81.22	80.27	80.54
	DL-CRoP	99.27	100	99.13
Case C	SVM	80.24	82.56	82.94
	KNN	76.75	77.32	75.24
	AdaBoost	84.73	85.31	86.22
	Random forest	72.35	74.47	74.02
	Naïve Bayes	76.12	75.82	77.92
	DL-CRoP	93.21	92.54	92.69
Case D	SVM	56.05	57.25	57.80
	KNN	52.11	52.96	53.04
	AdaBoost	62.37	64.12	62.18
	Random forest	50.08	51.84	52.20
	Naïve Bayes	53.28	54.70	53.13
	DL-CRoP	66.91	68.54	68.04

## Discussion

ML has revolutionized animal and plant sciences the world over [6–8,10–17]. In the field of ML, DL is emerging as a better platform for analyzing image-based detection of traits of commercial, agronomical, and pathological importance [6–8,10–17]. The CNN algorithms have been successfully used in animal behavioral studies, for instance, shark territorial behavior [20], prairie dog behavior mimicked by the algorithm [21], and the foraging behavior of dwarf mongooses [22]. More interestingly, gazelle optimization algorithm could predict the behavior of gazelle feeding in the safe, i.e., exploration state, and evading away from predators, i.e., exploitation situations [23]. The natural eruption pattern of geysers could be predicted with the geyser-inspired algorithm, which could predict the eruption pattern of geysers along with exploration and exploitation of search spaces [24]. Among several algorithms of DL, the CNN represents a precisely designed algorithm for image processing and recognition tasks. The CNN-based DL platforms are widely used for the detection of several disease-related markers in plants and human diseases [6–8,10–17]. The DL framework BRBFNN (a bacterial foraging-optimization-radial basis function neural network) is successfully used for identifying and classifying plant leaf diseases [25]. The region growing algorithm (RGA) was used to perform feature extraction. Bacterial foraging optimization (BFO) and radial-based function neural network (RBFNN) consisting of hidden, input, and output layers were applied for framework training. The framework had a better average specificity of 0.8558 compared to K-means (0.7914) and genetic algorithm (0.8139) [26]. The N status of wheat leaves grown in the field conditions has been successfully predicted using a genetic algorithm and neural network fusion [26]. The framework was able to achieve accuracy rates ranging from 2.73 to 16.56 for different combinations of image corrections for different levels of mean absolute percentage error [26]. Nutrient deficiencies in black gram, specifically under N, K, iron, magnesium, and calcium, have been identified [27]. The analysis used 2 separate datasets: a training dataset and a test dataset, each containing 30 images of both healthy (control) and nutrient-deficient leaves. The color-based detection of nutrient deficiency was a difficult challenge, and the method developed could partially achieve a successful interpretation of the image with 90% accuracy [28]. The detection of plant diseases through the CNN application is a daunting task. Two pretrained models of CNN, namely, Visual Geometry Group (VGG16) and VGG19, could successfully detect the diseased leaves of maize plants from the healthy ones [29]. These models used the orthogonal learning particle swarm optimization algorithm coupled to a rate of exponential decay learning compared to traditional manual trial and error methods. The proposed model achieved 98.2% accuracy compared to Xception (96.5%) and Inception-v3 (96.6%) for the same maize leaf dataset [29]. Application of VGG16 and InceptionV3 for rice diseases and pests using CNN has been successfully carried out [30]. Rice 1426 image dataset with 9 classes, namely, Hispa, Sheath Blight, Bacterial Leaf Blight, Neck Blast, False Smut, Brown Plant Hopper, Stemborer, Sheath Rot, and Brown Spot, was used for the framework. The proposed CNN, i.e., simple CNN, was able to achieve 94.33% mean validation accuracy compared to other CNN architectures [30]. Previously, crop defective ranking was used for measuring the defective pixel density of leaf images, which differ under nutrient-deficient conditions. The method was able to use leaf images to detect the deficiency symptoms of nitrogen, phosphorous, and potassium in paddy leaves with a reasonable average accuracy of 90% [31]. The application of CNN for species-level identification in plants needs more accuracy. Compared to other CNN models, DL-CRoP was able to attain a satisfactory accuracy level in the species-level identification of up to 90.45% using the leaf image dataset of 3 different species (tomato, maize, and Vigna) (Tables 1 and 2). Similarly, the application of stem images for the precision identification of crop species is limited. The proposed DL-CRoP has been able to achieve the highest value, i.e., 1 for recall, precision, and F1 score (Tables 1 and 2). Compared to other algorithms like SVM, KNN, AdaBoost, naïve Bayes, and random forest, DL-CRoP had a higher level of accuracy in stem-based species detection at 80–20, 70–30, and 60–40 splits (Table 2). Nutrient-deficient conditions in the leaf of black gram subjected to control, calcium, iron, magnesium, nitrogen, potassium, and phosphorus deficiencies have been studied using ML. The CNN model called ResNet50 was able to achieve an overall good level of precision (68.01%), recall (64.39%), and F measure (66.15%) for the image dataset used. For instance, the precision (%), recall (%), and F measures were 57.40%, 74.40%, and 64.80% for calcium, 83.76%, 70.10%, and 76.32% for iron, 65.88%, 60.54%, and 63.09% for potassium, 78.76%, 61.82%, and 69.26% for magnesium, 73.68%, 34.56%, and 47.05% for nitrogen, and 70.20%, 73.93%, and 72.01% for phosphorus [32]. Detection of calcium and potassium deficiency in tomato fruits can be estimated with InceptionResNetV2-based CNN. The CNN architecture could detect calcium and potassium deficiency in tomato fruits with 92.5% and 87.5% test accuracy [33]. Early detection of nutrient deficiency in maize leaves is a matter of agricultural importance for a farmer. It will give ample time for farmers to overcome the deficiency of a specific nutrient through fertilizer application. In this context, DL-CRoP was able to detect nitrogen deficiency in maize leaves with 93% accuracy, a much higher accuracy achieved compared to other established CNN modules (Tables 1 and 2). Root-based image datasets are lesser explored for CNN-based prediction of plant health status. Our DL-CRoP used the maize root image datasets of plants grown under normal and nitrogen-deficient conditions, and the algorithm was able to detect the nitrogen deficiency in maize roots at an accuracy level of 68.54%, much higher compared to SVM, KNN, AdaBoost, random forest, and naïve Bayes (Table 2). From the current study, DL-CRoP precision and accuracy are highly satisfactory compared to CNN algorithms in use [34–36]. The DL-CRoP performance on different case studies showed superior performance than other algorithms tested. The results showed the capability of DL-CRoP to classify efficiently the class type from different plants or the same plant under different conditions. Furthermore, the simple outplay and the algorithm used for case studies A to D also indicate the robustness of DL-CRoP in predicting the species-level identification using stem and leaf images and nutrient deficiency conditions with the highest level of precision and accuracy (Figs. 1 to 5). This model can be utilized to create a real-time, embedded image-based system for detecting abiotic stresses in crops along with the onset and severity of stress levels. It allows for the timely triggering of rescue actuators through mobile-friendly applications, enabling farmers to respond promptly from remote locations. DL-CRoP with its robustness in terms of precision and accuracy has relevance in practical agriculture. For instance, leaf nitrogen deficiency symptoms can be easily detected using the DL-CRoP platform. The early diagnosis of nitrogen deficiency in the leaf of a plant is of vital significance in timely addressing the issue of nutrient deficiency using appropriate fertilizer treatment. Furthermore, MHA use has improved the diagnosis ability of DL-CRoP in a scenario when leaf images may show a mosaic pattern of nitrogen deficiency compared to a more localized symptom (Fig. 6). In the future, DL-CRoP can be integrated into existing farming practices as an Android app where a leaf image will be analyzed for its nutrient health status. DL-CRoP is trained on the nitrogen deficiency symptoms of maize leaf and the concept can be extended to other nutrient deficiency conditions in other crops as well. For instance, the DL-CRoP platform can be further used for the identification of different species using leaf and stem image datasets. In particular, the application of DL-CRoP in diagnosing the nitrogen deficiency in maize leaf can be extended to cash crops such as tomato, lettuce, and other horticultural crops. Similarly, DL-CRoP can also be trained on other macronutrient (phosphorus, potassium, and magnesium) and micronutrient (zinc and copper) deficiency symptoms.

## Materials and Methods

ML is an exciting platform to develop automated systems. These systems can be used in identifying different species of crops and weeds. It provides an alternative method to use herbicides to kill unwanted plants. Recent advancements in the ML approaches enable us to identify plants using their images, although the image-based classification posed challenges related to interrelated species similarity, intra-class diversities, background effects, color, and illumination variations available in the image datasets. Prior work employed different types of supervised learning algorithms in support of handcraft features and global features for investigating plant identification [37,38]. The CNN-based DL approach called DL-CRoP in precise plant-level species identification and detecting a nutrient deficiency in a crop plant is the least explored to date. Keeping that in mind, the present study used a deep CNN approach in the automation and recognition of the joint feature of plant phenotype images.

### Case studies, growth conditions, image acquisition, and datasets

The proposed work has an objective to develop a CNN model for correct and high-precision identification of species using shoot and leaf image datasets. Further, to identify the health status of a plant using leaf and root image datasets, the entire CNN framework was conducted on 4 case studies: case A: shoot identification, case B: leaf identification, case C: leaf health status, and case D: root health status. Among these, case studies A and B were used to create shoot and leaf image datasets to train the CNN model for correctly identifying a species, i.e., maize, tomato, and Vigna. In contrast, case studies C and D were conducted to develop leaf and root image datasets for the CNN framework to identify the nitrogen (N) deficiency in maize in the leaf and root system. The plants of maize, tomato, and Vigna were sown in pots containing soil (garden soil:farmyard manure, 3:1) for 60 d (Fig. S1).

### Experimental validation of DL-CRoP platform

The Jammu University-Botany Image Database (JU-BID) was constructed using the on-field experimentation carried out at the Botany Department, Jammu University, India (32.72°N, 74.85°E). The seeds were surface sterilized with 2% hypochlorite for 10 min, followed by 10 min in 70% ethanol, and washed 3 times with deionized water before sowing. Each species’ nursery bed was prepared, and healthy seedlings were picked and transported to 25*26 pots filled with 10- to 12-kg soil. Pots were supplied with routine irrigation based on the needs of the plant type. Variables like temperature, relative humidity, and light were natural.

#### Preparation of shoot dataset (case study A)

The database JU-BID comprised of a collection of shoot images of maize, tomato, and Vigna grown in pots in the botanical garden of the Botany Department, Jammu University during the kharif season of 2019–2023. There were 50 pots of each class every year; thus, the total pot per class in 3 years was 150. The imaging system comprised an automated prototype, lighting system, and camera. The pot was placed on an automated prototype platform to capture images of shoots using a digital single-lens reflex (DSLR) (Canon EOS3000 D) camera as shown in Fig. 4. The camera offered a higher resolution with 16 megapixels. It consisted of a Canon DIGIC4 image processor with a 28-mm wide-angle lens and a shutter speed of 1 to 1/3,200 s. It had a 1/2.3-inch CCD sensor having 1,600 maximum ISO sensitivity and an aperture of dimensions f/3.5-f/5.9. A SUNPAK 200I UT (tripod stand) was used to stabilize and support the camera. Every 5 s, the automated platform rotates 30° followed by image capture. A total of 12 images at different angles (30, 60, 90, 120, 150, 180, 210, 240, 270, 300, 330, and 360) were captured from a single plant of each class. After one round of imaging, the time lapse was automatically increased to 2 min, termed as stationary phase or preparatory phase. During this phase, another pot of the same class or a different class was arranged for imaging. The quality of the scanned image was of dimensions 2,478*2,286 and resolution 300 dpi. A few images of 3 classes with color images with 1-ft/pixel resolution are shown in Fig. S1A. The dataset covered 500, 450, and 650 images in each class, creating a dataset of 1,600 shoot images.

#### Preparation of leaf dataset (case study B)

The dataset JU-BID contains 3 classes of leaves, namely, maize, tomato, and Vigna. The leaves were harvested from plants at different stages of growth followed by scanning at 600 dpi using a scanner (Epson Perfection V850 Pro Scanner). The quality of scanned images, namely, dimensions (544*871 pixels) and resolution (300 dpi), and a few scanned images of 3 classes with 256 × 256 color images with 1-ft/pixel resolution were illustrated in Fig. S1B,C and D.

#### Preparation of dataset (case study C)

The dataset was 2 different conditions of maize leaves, namely, one class has nitrogen-deficient leaves (N−), and another class has normal nitrogen leaves (control, N+). Plants grown in normal nitrogen (control) contained 2.5 mmol Ca(NO_3_)_2_, 0.75 mmol K_2_SO_4_, 0.25 mmol KH_2_PO_4_, 0.13 mmol EDTA-Fe, 1.0 mol ZnSO_4_, 1.0 mol H_3_BO_3_, 1.0 mol MnSO_4_, 0.1 mol CuSO_4_, and 0.005 mol (NH_4_)6Mo_7_O_24_. The nitrogen deficiency (N- solution) contained N (0.025 mmol of CaNO_3_), and the Ca^2+^ concentration was adjusted with CaCl_2_ to the same level as the control. The other nutrient concentrations remained the same as in the control. The leaves from each treatment were harvested, and in all, 800 images were acquired by scanning leaves at 600 dpi (Fig. S1E). It represents leaf images of 2 classes of color images with 1-ft/pixel resolution.

#### Preparation of dataset (case study D)

The dataset was 2 different conditions of maize roots, namely, one class has nitrogen deficiency root (N−), and another class has normal nitrogen root (control). The details of both the treatments remained the same as in case study C. About 20 d after treatment, roots of each class were harvested and images were scanned at 600 dpi (Fig. S1F).

The dataset of each class was stored with the name of the respective class, i.e., cases A and B (maize, tomato, and Vigna) and cases C and D (N− and N+ control) followed by splitting into 3 groups: training, testing, and cross-validation. Specifically, the DL-CRoP training was carried out on 80% of the images, cross-validation on 10%, and 10% for testing. This ratio of 80:10:10 was similar for every class in the model. JU-BID comprised image datasets for the following cases: case study A (maize, tomato, and Vigna, 1,000 images each), case study B (maize, tomato, and Vigna, 650 images each), case study C (nitrogen in normal concentration and nitrogen in deficient conditions in maize leaf, 400 images each), and case study D (normal nitrogen and nitrogen deficiency in maize roots, 400 images each). Three different species, i.e., *S. lycopersicum* L. (tomato), *Z. mays* L. (maize), and *V. radiata* (L.) R. Wilczek (Vigna), were studied for case studies A and B.

### Working module

A DL-based crop species and plant health identification CNN framework called DL-CRoP has been constructed. The details of the CNN architecture included 42 layers deep and used InceptionResNetV2 as a backbone network for the feature extraction process and enabled the identification of various plant species, i.e., maize, tomato, and Vigna (mung bean). InceptionResNetV2 is a well-trained CNN trained on several million image datasets, although other alternatives exist like InceptionResNet, Inception v3, Inception v4, Inception v1, and Inception v2. InceptionResNetV2 was preferred due to its highest training power and better accuracy. The InceptionResNetV2 network of layers was subjected to regularized implementation as an auxiliary classification model. InceptionResNetV2 delivered top-of-the-line precision with top 1 and top 5 error rates of 19.9% and 4.9%, respectively. DL-CRoP was trained on the novel plant image dataset named JU-BID and was constructed under a controlled environment at the University of Jammu during the Kharif season of 2019–2023. The image datasets used for the DL-CRoP training are freely available at GitHub (https://github.com/urfanbutt) in the form of case studies A to D.

### DL-CRoP platform overview

The DL-CRoP architecture and training methodology have been thoroughly outlined (Figs. 4 and 5). The model begins with the pipelining dataset construction phase and concludes with forecasting the model’s estimation. The data augmentation techniques provided a balanced number of images for all classes. Furthermore, by incorporating some augmented images in the training dataset, data augmentation strategies could expand the dataset size and minimize overfitting while the model was being trained. Additionally, while constructing a primary dataset, noisy elements and color imperfections were also captured during data acquisition.

#### Image dataset creation

Initially, the image datasets of case studies A to D were collected by using a DSLR camera and a scanner. The images of these datasets were stored in the data cloud, which was then processed with a deep lens followed by data cleaning in the preprocessing phase, a primary step in the proposed framework (Figs. 4 and 5). The image rescaling, color correction, data augmentation, and noise reduction were performed on the JU-BID datasets. The color correction was performed by principal components analysis (PCA) color augmentation, which modified the color intensity values of the pixels by normalization. The augmented processed images were resized to 1,024 x 1,024, and then further added as input to the InceptionResNetV2 network.

Transfer learning with the InceptionResNetV2 model was performed, where convolutional layers stand out as critical for feature extraction. They were the backbone of the process, capturing intricate patterns from the input data using the following equation:

$$Y_{i,j}=\sum_{m,n}X_{i+m,j+n}\times W_{m,n}+b$$

where *Y*_*i*,*j*_ shows the map output feature, *X*_*i* + *m*,*j* + *n*_ is the map input feature, *W*_*m*,*n*_ denotes the convolutional filter/kernel, and *b* signifies the bias term. These layers leverage pre-learned knowledge from a vast dataset, improving the model’s ability to understand complex data. The Inception module takes things further by incorporating parallel convolutional filters and residual connections, as shown by the following equation:

$$Y=F\left(X\right)+X$$

To tackle the challenge of training deep networks, pooling layers play an essential role in down-sampling feature maps while preserving essential information, characterized by the following operation:

$$Y_{i,j}=\max_{m,n}X_{i+m,j+n}$$

Meanwhile, the rectilinear unit (ReLU) activation function, *f*(*x*) =  max (0, *x*), shows nonlinearity, allowing the model to capture intricate patterns. Using the power of transfer learning, the InceptionResNetV2 model showcases exceptional performance across various computer vision tasks, leveraging insights from pretrained models to excel even with limited labeled data. With a network of 164 layers, the InceptionResNetV2 network concatenated Inception modules with residual connections for learning in-depth features and reduced the effect of the vanishing gradient problem. The network also included the reduction blocks to optimally utilize the spatial space by selectively apprehending abstract features that carried more detailed information about the image. Further stacking of 2 fully connected layers was attached to improve the model’s accuracy rate by providing detailed relativity between the features to be learned.

 The images along with their true labels function are the basis for model training. Employing non-maximum suppression (NMS) for every meta-architecture, the primary objective of training the DL-CRoP model was to minimize the errors between the final and estimated results and to mitigate losses between the final results and actual outcomes. Transfer learning was performed on the InceptionResNetV2 network with freezing at the lower layers. For each convolution layer (conv), there was a filter expansion block of 1 × 1 conv with the ReLU layer. The batch normalization was applied to the top layer architecture. The intermediate features were extracted during the initial convolution and max pooling layers (Figs. 4 and 5). For training DL-CRoP, all the filters in the convolution layers were concatenated with each other using eigen values *e_i_* to yield a sequence of unified extracted features. For retaining the channel dimensions, max pooling along with a flattened layer was added in the proposed model. This addition reduced the spatial dimensionality of the mapped pooled features.

The features extracted by the convolution layer were then subsequently passed to a 2-layer-based fully connected layer so that maximum features could be absorbed by the network. It improvised the input to be given to the Softmax layer, which consequently generated a probabilistic prediction value for each class *C_i_*. The outcome was a predicted value of class *C_i_* with maximum probability for each case study in JU-BID. Further back propagation was applied to adjust hyperparameter values to minimize the cross-entropy loss function. In this case, mean square error (MSE) assessed the discrepancy degree between the predicted and actual values. Likewise, a stochastic gradient descent optimizer with RMSProp (root mean square propagation, an innovative stochastic mini-batch learning method) and sparse cross entropy were used as an inbuilt objective function over mini-batches for crop plant species and health identification. The designed proposed framework DL-CRoP could further be extended for use in other plant species identification as well.

### DL-CRoP training

For model training, images and their corresponding ground-truth labels were fed to the classifier to minimize the error of final results using the NMS of each meta-architecture. The hyperparameters were initialized with stochastic gradient descent along with RMSProp at a batch size of 10 with an initial 0.001 learning rate, 0.9 momentum, and decaying rates of 0.0005 with β_1_ = 0.9 and β_2_ = 0.999, ρ = 0.9, and ε = e^−7^, respectively. These parameters were selected through multiple initial trials based on the design of Sutskever et al. [39]. The error function was employed to evaluate the performance of various networks on a new dataset that was independent of those used for training. Understanding how an algorithm’s execution time varied in response to the amount of input data was possible through knowledge of its temporal time complexity. The rationale underlying the Inception model was CNN’s convergence time given by O (*S^2^. K^2^. C_IN_. C_OUT_*), where S indicates the spatial length of the kernels, K is the kernel size, and *C_IN_* and *C_OUT_* indicate the input and output unit’s numbers, respectively. Since InceptionResNetV2 could extract high-level characteristics from the image and exclude the redundant information, the proposed framework had high computational complexity yet performed well in terms of space complexity with spatial aggregation on lower dimensions.

### Evaluation metrics

The performance indicators like accuracy, recall, precision, and F1 score outcomes for TP, FP, TN, and FN were used to analyze the results. The percentage of correctly classified image classes to all the classes in a dataset defined the accuracy given by the following equation:

$$\begin{array}{c} \text{Accuracy}=No.\;\text{of correct images classified}/\\ \;\text{Total number of predictions} \end{array}$$ (1)

Moreover, the correct and incorrect predictions were specifically explained using TP, which represents the number of correctly categorized positive images, TN, which denotes the number of accurately recognized negative images, FP, which indicates that the image has been predicted to be positive, although the image was negative, and FN, which represents an image belonging to a positive class but has been predicted negative. FP and FN specify type I and type II errors, indicating misclassified image instances in the class. Hence, accuracy could be more specifically represented as

$$\text{Accuracy}=\frac{TP+TN\;}{TP+TN+FP+FN}\;$$ (2)

The performance parameter precision indicated the proportion accurately expected positive images to accurately all predicted positive images given by the equation

$$\text{Precision}=\frac{TP}{TP+FP}\;$$ (3)

Recall, on the other hand, defines the accurately predicted positive image percentage to all images falling under the corresponding class as shown by the following equation.

$$\text{Recall}=\frac{TP}{TP+FN}\;$$ (4)

The harmonic means of precision and recall values indicate the F1 score given by Eq. 5.

$$F1\;\text{score}=\frac{2\times \text{Precision}\times \text{Recall}}{\text{Precision}+\text{Recall}}\;$$ (5)

The confusion matrix was used to summarize performance of DL-CRoP on the provided dataset (JU-BID). It represented the number of accurate and inaccurate instances based on DL-CRoP model prediction. The confusion matrix provided the number of instances produced by DL-CRoP on JU-BID.

TP: The model could correctly predict a positive outcome.

TN: The model could correctly predict a negative outcome.

FP: The model could incorrectly predict a positive outcome (type I error).

FN: The model could incorrectly predict a negative outcome (type II error).

The TP, TN, FP, and FN values were obtained using a confusion matrix for any ML/DL-based model. Thus, the confusion matrix illustrated an analysis of the output class and target class into a 2-dimensional matrix structure, where the output class reflected the learning model’s prediction result and the target image class represented the ground-truth class of an image [18].

### Comparison of DL-CRoP with other algorithms

DL-CRoP was compared with other algorithms such as naïve Bayes, AdaBoost, KNN, random forest, and SVM for the same JU-BID datasets. The classification accuracy of the proposed DL-CRoP with the algorithms mentioned above was determined at 80:20, 70:30, and 60:40 split ratios.

### MHA analysis for nutrient deficiency diagnosis

In the proposed platform of DL-CRoP, MHA is included before 3 fully connected layers for investigating the enhancement of diagnosis of nitrogen deficiency in the leaf image dataset (case study C). In brief, MHA is a technique in DL that permits the model to focus on specific parts of the leaf images for a reliable prediction. The MHA and VGG-16 structures have been trained to capture comprehensive hierarchical representations of leaf image features, resulting in precise and accurate prediction of nitrogen deficiency. VGG-16 is considered advanced MHA due to its depth, with 16 layers in total, including 3 fully connected layers and 13 convolutional layers [40]. The novelty and uniqueness of DL-CRoP are based on the attributes of using InceptionResNetV2 for diagnosing leaf and root nitrogen deficiency. Furthermore, MHA analysis also improves the accuracy of DL-CRoP in diagnosing leaf nitrogen deficiency symptoms.So far, computational biology has been successfully used for the identification of transcription factor (TF) pairs regulating a gene of interest in plants. The application of MHA in the computational biology of plants offers a promising avenue in the near future [41].

## Data Availability

The JU-BID image database is available at https://github.com/urfanbutt, while the algorithm is available in the Supplementary Materials.

## Supplementary Materials

Supplementary 1 Fig. S1 Algorithm
