# Supplementary material for: The Deep Learning-Crop Platform (DL-CRoP): For Species-Level Identification and Nutrient Status of Agricultural Crops
Source: Research (Wash D C). 2024 Oct 4;7:0491. doi: 10.34133/research.0491 (PMC11450475; doi:10.34133/research.0491)
Supplement: Supplementary 1 — Fig. S1 Algorithm [file research.0491.f1.docx]

**Title:**

The Deep Learning-Crop Platform (DL-CRoP): For Species Level Identification and Nutrient Status of Agricultural Crops

**Short title:** Deep Learning for Crop Health

Mohammad Urfan^1^, Prakriti Rajput^1^, Palak Mahajan^2^, Shubham Sharma^1^, Haroon Rashid Hakla^1^, Verasis Kour^3^, Bhubneshwari Khajuria^1^, Rehana Chowdhary^1^, Parveen Kumar Lehana^3^, Namrata Karlupia^4^, Pawanesh Abrol^4^, Lam Son Phan Tran^5*^ and Sikander PAL Choudhary^1^*

^1^Crop Physiology Laboratory, Department of Botany, University of Jammu, Jammu 180006, INDIA

^2^Department of Computer Science & Engineering, Central University of Jammu, Jammu 181143, INDIA

^3^Department of Electronics, University of Jammu, Jammu 180006, INDIA

^4^Department of Computer Science & IT, University of Jammu, Jammu 180006, INDIA

^5^Institute of Genomics for Crop Abiotic Stress Tolerance, Department of Plant and Soil Science, Texas Tech University, Lubbock, TX, 79409, USA

*Address correspondence to:

Lam Son Phan Tran: [son.tran@ttu.edu](mailto:son.tran@ttu.edu)

<https://orcid.org/0000-0001-9883-9768>

Or

Sikander PAL: [sikanderpal@jammuuniversity.ac.in](mailto:sikanderpal@jammuuniversity.ac.in)

<http://orcid.org/0000-0002-3827-466X>

**Supplementary data:**


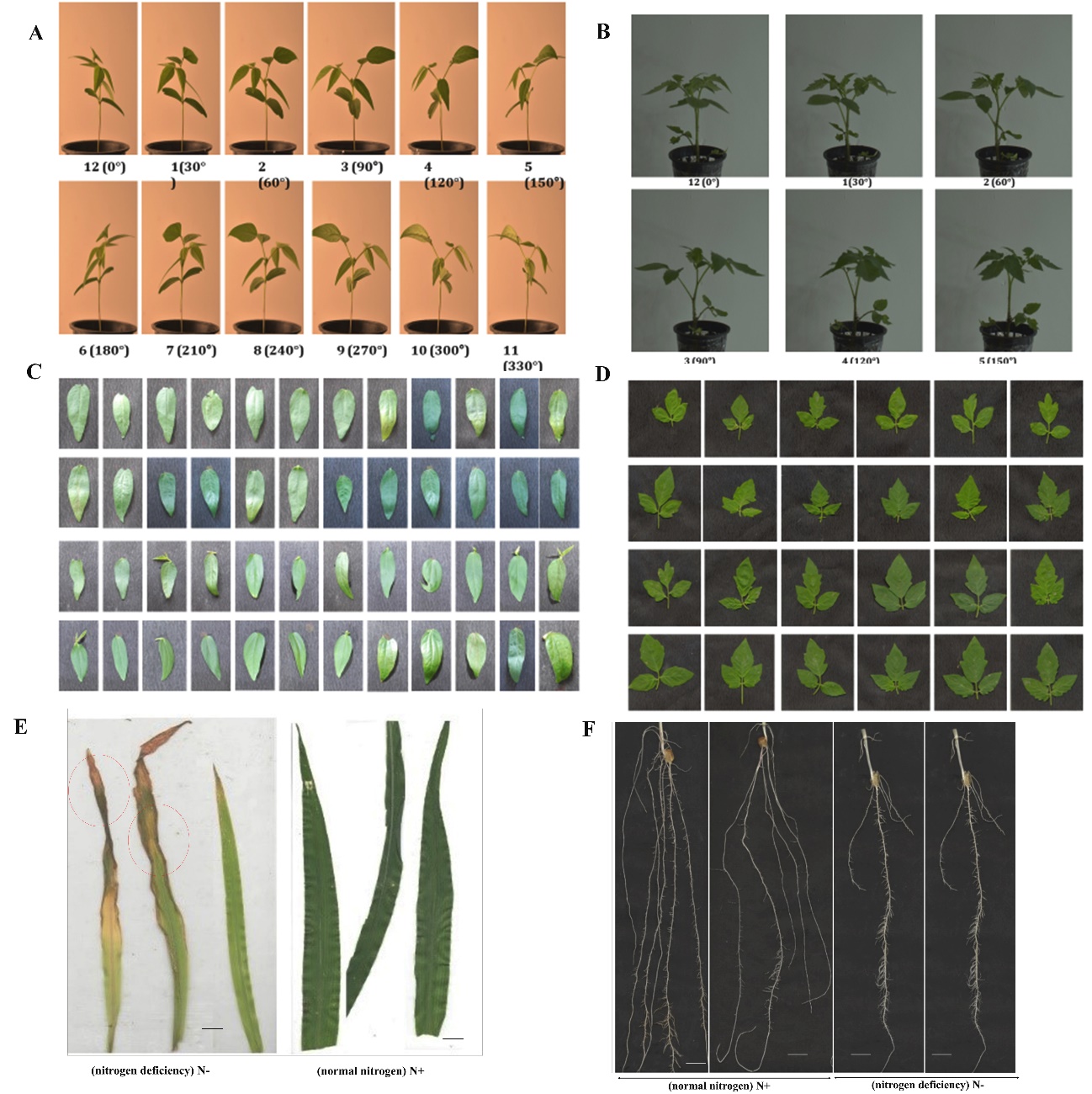


**Fig. S1. The Jammu University-Botany Image Database (JU-BID) of case studies.**

comprised a collection of stem images of maize, tomato, and Vigna plants grown in pots. For Case Study A: the dataset covered 500, 450, and 650 images in each class, creating a dataset of 1600 stem images (A); for Case Study B: comprised leaves of maize (B), tomato (C), and Vigna (D) plants at different stages of growth followed by scanning at 600 dpi; For case study C: (E) data set included maize leaves of plants subjected to high (N+) and low nitrogen (N-) conditions; the case study D: (F) comprised of maize roots plants subjected to normal nitrogen (N+) and nitrogen deficiency (N-).

Algorithm code used:

imds=imageDatastore('D:\cnn', 'IncludeSubfolders',true, ...

'LabelSource','foldernames');

[imdsTrain,imdsValidation] = splitEachLabel(imds,0.7,'randomized');

net = inceptionresnetv2;

lgraph = layerGraph(net);

figure('Units','normalized','Position',[0.1 0.1 0.8 0.8]);

plot(lgraph)

net.Layers(1)

inputSize = net.Layers(1).InputSize;

net.Layers

lgraph = removeLayers(lgraph, {'predictions','predictions_softmax','ClassificationLayer_predictions'});

numClasses = numel(categories(imdsTrain.Labels));

newLayers = [

fullyConnectedLayer(numClasses,'Name','fc','WeightLearnRateFactor',10,'BiasLearnRateFactor',10)

softmaxLayer('Name','softmax')

classificationLayer('Name','classoutput')];

lgraph = addLayers(lgraph,newLayers);

lgraph = connectLayers(lgraph,'avg_pool','fc');

figure('Units','normalized','Position',[0.3 0.3 0.4 0.4]);

plot(lgraph)

ylim([0,10])

layers = lgraph.Layers;

connections = lgraph.Connections;

layers(1:110) = freezeWeights(layers(1:110));

lgraph = createLgraphUsingConnections(layers,connections);

pixelRange = [-30 30];

imageAugmenter = imageDataAugmenter( ...

'RandXReflection',true, ...

'RandXTranslation',pixelRange, ...

'RandYTranslation',pixelRange);

augimdsTrain = augmentedImageDatastore(inputSize(1:2),imdsTrain, ...

'DataAugmentation',imageAugmenter);

augimdsValidation = augmentedImageDatastore(inputSize(1:2),imdsValidation);

options = trainingOptions('sgdm', ...

'MiniBatchSize',10, ...

'MaxEpochs',50, ...

'InitialLearnRate',1e-4, ...

'ValidationData',augimdsValidation, ...

'ValidationFrequency',3, ...

'ValidationPatience',Inf, ...

'Verbose',false ,...

'Plots','training-progress');

net = trainNetwork(augimdsTrain,lgraph,options);

[YPred,probs] = classify(net,augimdsValidation);

accuracy = mean(YPred == imdsValidation.Labels)

YTest=imdsValidation.Labels;

plotconfusion(YTest,YPred)

[YPred,scores] = classify(net,augimdsValidation);

idx = randperm(numel(imdsValidation.Files),4);

figure

for i = 1:4

subplot(2,2,i)

I = readimage(imdsValidation,idx(i));

imshow(I)

label = YPred(idx(i));

title(string(label));

end

% confusion matrix

figure

cm=confusionchart(YTest,YPred)

cm.ColumnSummary = 'column-normalized';

cm.RowSummary = 'row-normalized';

cm.Title = ' Confusion Matrix';

[m,order]=confusionmat(YTest,YPred);

Diagonal=diag(m);

sum_rows=sum(m,2);

Precision=Diagonal./sum_rows;

Overall_Precision=mean(Precision)

sum_col=sum(m,1);

recall=Diagonal./sum_col';

overall_recall=mean(recall)

F1_Score=2*((Overall_Precision*overall_recall)/(Overall_Precision+overall_recall))

idx = randperm(numel(imdsValidation.Files),9);

figure

for i = 1:9

subplot(3,3,i)

I = readimage(imdsValidation,idx(i));

imshow(I)

label = YPred(idx(i));

title(string(label));

end
